# Analysis of soil bacteria susceptibility to manufactured nanoparticles via data visualization

**DOI:** 10.3762/bjnano.6.166

**Published:** 2015-07-28

**Authors:** Rong Liu, Yuan Ge, Patricia A Holden, Yoram Cohen

**Affiliations:** 1 Center for Environmental Implications of Nanotechnology, University of California, United States; 2 Institute of the Environment and Sustainability, University of California, Los Angeles, United States; 3 Earth Research Institute, University of California, Santa Barbara, United States; 4 Bren School of Environmental Science and Management, University of California, Santa Barbara, United States; 5 Chemical and Biomolecular Engineering Department, University of California, Los Angeles, United States

**Keywords:** environmental impact, manufactured nanoparticles, nanoinformatics, soil bacteria, visualization

## Abstract

The impact of ZnO and TiO_2_ manufactured nanoparticles (MNPs) on soil bacterial communities for different exposure periods and MNP doses was explored via data visualization techniques. Interrelationships between MNP treatments and responses of bacterial taxa were illustrated by bipartite graphs, allowing fast identification of important soil bacterial taxa that are susceptible to MNPs. Contribution biplots with subcompositional coherence property were generated via log-ratio analysis (LRA), which jointly display the treatment distribution and the variance (contribution) of bacterial taxa. The LRA contribution biplots and nonmetric multi-dimensional scaling (NMDS) of the dataset, along with hierarchical clustering, demonstrated that high doses of ZnO and TiO_2_ MNPs caused significant compositional changes in soil bacterial communities. The suitability of family level for MNP taxonomic impact assessment was demonstrated by both the LRA biplots and simplified NMDSs with quantification provided by the distance correlation between MNP impacts summarized at different taxonomic levels. The present study demonstrates that visual exploration could potentially assist in knowledge discovery and interpretation of data on soil bacterial communities exposed to MNPs and thus evaluate the potential for environmental impacts.

## Introduction

Manufactured nanoparticles (MNPs) are now routinely used in numerous products and applications due to their novel functional properties that arise at the nanoscale [1–2]. However, as the applications of MNPs rapidly expand [2–3], there is an increased public concern regarding the potential environmental and health risks associated with MNPs [4–9] throughout their lifecycle [10–14]. MNPs may be released to the environment as the result of a variety of human-related activities (air emissions and/or direct discharge to surface water, etc.), wherein they can move across environmental boundaries and are therefore likely to be found in most media [13–14]. The presence of MNPs in the environment could lead to exposures of ecological receptors to MNPs via multiple pathways [13]. Although there is lack of field monitoring data regarding environmental concentrations for most MNPs, various simulations [14–15] of multimedia environmental distributions of MNPs suggest that MNPs tend to accumulate in soil and sediment [16–17]. Various studies [18–22] have reported that MNPs could lead to adverse environmental impacts. For example, Ag and Pt MNPs may interfere with zebrafish embryo hatching [23]; ZnO MNPs may cause compositional changes in soil bacterial communities [18–19]; quantum dots (QDs) were linked to DNA damage of both freshwater mussels and gills [24]; and carbon nanotubes have been found to induce harmful effects to various organs (such as aquatic animals, bacteria, and plants) [25].

MNPs in soil can cause compositional changes to soil bacterial communities and thus may induce profound impacts on terrestrial ecosystems [16,26]. Soil microbial communities, as one of the most abundant and diverse groups of organisms on earth, perform many critical ecosystem functions (e.g., element cycling and waste decomposition) [27–28] and are important biotic indicators of soil health [29]. Therefore, information about MNP effects on soil microbial communities is critical for environmental impact assessment [13]. Recently, efforts [18–19,26,30–31] have been devoted to investigate the impacts of various MNPs on soil bacterial communities, resulting in large datasets of high dimensionality (e.g., over 10^5^ soil DNA sequences extracted for a treatment) [18–19]. Therefore, advanced data exploration/visualization approaches are required to allow researchers to design subsequent confirmatory experiments and/or perform detailed statistical analyses. Graphical displays of multivariate (high-dimensional) ecological data can also facilitate data comparison and interpretation (e.g., acquainting variables of important roles/contributions and identifying similarity/distribution among samples) [32]. In addition, since bacterial community data are usually compositional (each sample is profiled by a set of non-negative values that add up to unity), it is important that their analyses are subcompositionally coherent (i.e., the relationship between two components (variables) should be the same and not dependent on the presence/absence of other components) [32].

Accordingly, in the present work, we report on a range of visual exploration approaches suitable for analysis of high content dataset for bacterial communities exposed to MNPs. Bipartite graphs [33–35] were established to illustrate interrelationships between MNPs and responses of bacterial taxa. Log-ratio analysis [32,36–37] that has subcompositional coherence property was utilized to generate biplots for joint displays of sample (treatment) separation/distribution and the contribution of bacterial taxa (i.e., the variances of bacterial taxa across all the treatments). In addition, the impacts of different MNPs were projected and explored via two-dimensional (2D) maps constructed by hierarchical clustering [32,38–39] and multidimensional scaling [32,40]. Also, a recently developed distance correlation [41] was employed to quantify the consistency between MNP impacts summarized at a range of taxonomic levels.

## Materials and Methods

### Data for soil bacterial communities exposed to MNPs

Visual exploration was conducted for a previously reported dataset of MNP impacts on soil bacterial communities [18]. The dataset contained 15 treatments (i.e., different MNP exposure tests) including TiO_2_ and ZnO MNPs of primary size in the range of about 15–20 nm and about 20–30 nm [42], respectively. The soil bacteria were exposed to the above MNPs for 15 and 60 days at three different doses (0.5, 1.0, and 2.0 mg/g (soil) for TiO_2_ MNPs and 0.05, 0.1, and 0.5 mg/g (soil) for ZnO MNPs) as well as 0, 15, and 60 day controls (without MNPs) [18]. Soil DNA sequences were recovered for the above 15 treatments (in quadruplicate). The recovered DNA sequences were clustered into 31,621 bacterial operational taxonomic units (OTUs) [18], with the number of DNA sequences clustered into the same OTU counted to quantify the impact of the 15 treatments on soil bacterial communities [18]. The OTUs were further summarized/assigned into a set of hierarchical taxa (i.e., genus (446), family (135), order (53), class (41), and phylum (19); the total number of taxa at each taxonomic level is given in the parentheses) [18]. For each taxonomic level (including OTU), the total counts of sequences assigned to a speciﬁc taxon represent its abundance, while the relative abundance of the taxon in the whole community was used as a measure of the impacts of the 15 treatments [18].

### Exploration workflow

**Figure 1:**
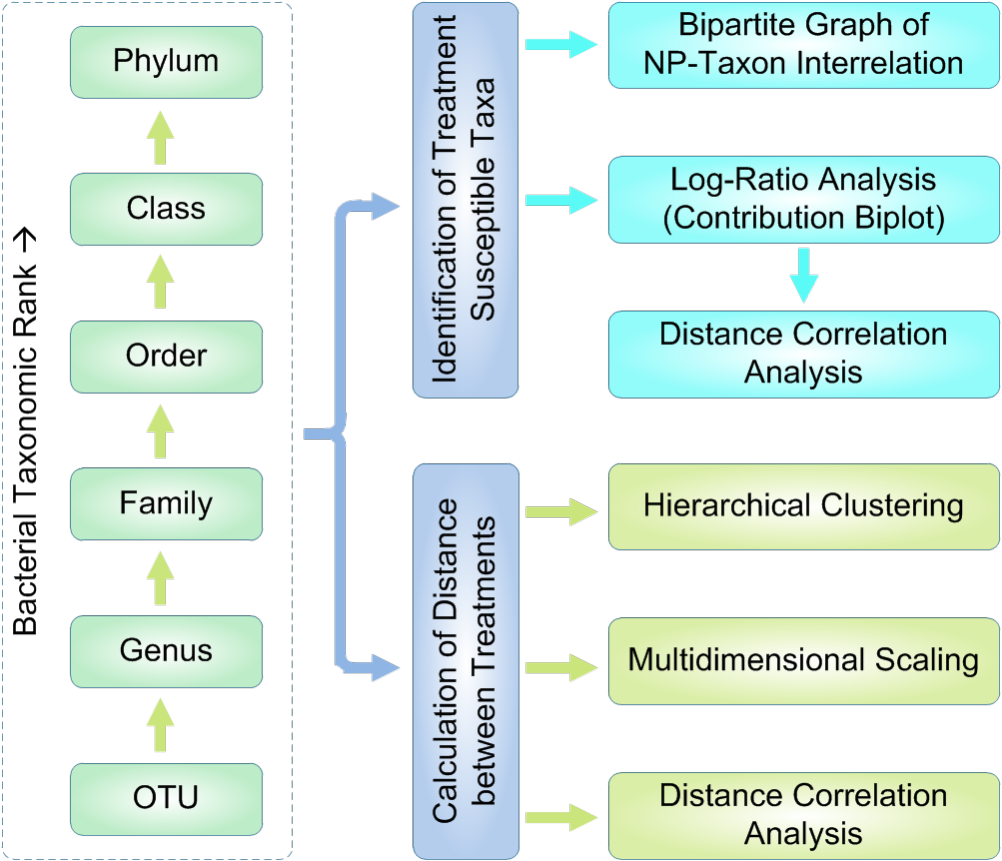
Workflow for visual data exploration of soil bacteria susceptible to MNP treatments.

Visual exploration of the above soil bacterial community data [18] followed a workflow summarized in Figure 1. The analysis was conducted to identify significant MNP-bacterial taxon interrelationships and to assess the similarity of MNP impacts on soil bacterial communities. For each taxonomic level (from genus to phylum), bacterial taxa that are susceptible to MNP treatments were identified according to a threshold of inter-percentile range. Interrelationships between the MNP treatments and the identified susceptible bacterial taxa were illustrated using bipartite graphs [33–35]. Biplots were generated by log-ratio analysis [32,36–37] (of subcompositional coherence property) to jointly display the separation (distribution) of treatments and the contribution (variance) of bacterial taxa. Multidimensional scaling analysis [32,40] was conducted, along with hierarchical clustering, in order to illustrate the main underlying structure of the soil bacterial community dataset. In addition, distance correlation coefficients [41] were calculated to assess the consistency of MNP impacts summarized at different taxonomic levels.

### MNP-Bacteria Interrelationships

The interrelationships between MNPs and the responses of bacterial taxa were explored using bipartite graphs [33–35]. It is noted that some bacterial taxa demonstrated only marginal variance across the 15 treatments (in quadruplicate), indicating their insusceptibility to the treatments. It is noted that the presence of treatment insusceptible bacterial taxa will complicate bipartite graphs without adding useful information. Therefore, in the present work, bacterial taxa which is in the 95th–5th percentile range in terms of relative abundance across all the 15 treatments (in quadruplicate) less than a prescribed threshold (e.g., 10/*n*, where *n* denotes the total number of bacterial taxa at a given taxonomic level) were discarded as being treatment insusceptible. The relative abundances of the remaining bacterial taxa that were considered as treatment susceptible were re-scaled to sum up to unity for each treatment. Bipartite graphs were then established based on the averaged relative abundance of bacterial taxa for each quadruplicated treatment. In an established bipartite graph, treatments and bacterial taxa were represented as nodes on opposite sides of the graph, with linkages between them indicating the bacterial taxa (and their relative abundance) identified for each treatment or vice versa.

### Log-ratio analysis

Log-ratio analysis (LRA) [32,36–37] was conducted for the bacterial taxa that were identified as treatment susceptible in order to further explore and visualize the impact of TiO_2_ and ZnO MNPs on the soil bacterial communities. In LRA, the relative abundances of bacterial taxa (i.e., compositional variables) were transformed to log-ratios to attain subcompositional coherence [32,36–37]. For example, given a dataset of four compositional variables (i.e., components) *a*, *b*, *c*, and *d*, a subcompositional dataset of *a’*, *b’*, and *c’* can be obtained by discarding component *d* (note that the subcompositional dataset is closed again, i.e., *a’* = *a* / (*a* + *b* + *c*), *b’* = *b* / (*a* + *b* + *c*), and *c’* = *c* / (*a* + *b* + *c*) so that *a’* + *b’* + *c’* = 1). After log-transformation, the distance between the composition *a’* and *b’* is given by:

[1]
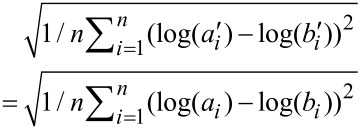


where *n* denotes the total number of samples in the dataset. It is noted that the log-ratio distance between two components remains the same irrespective of the presence/absence of other components (i.e., subcompositional coherence).

In LRA, once a compositional data matrix ***G*** (e.g., relative abundance of bacterial taxa) is transformed into log-ratios, a double centered matrix (i.e., row and column sums are all equal to zero) is constructed as:

[2]
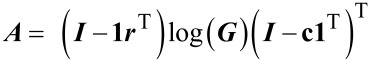


where ***I*** and **1** denotes identity matrix and vectors of ones of appropriate size, respectively. In addition, the two vectors ***r*** and ***c*** are the row and column sums of ***G*** relative to the grand total. The above double centered matrix is further weighted as follows:

[3]
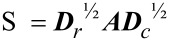


where ***D***_r_ and ***D***_c_ are the diagonal matrices corresponding to vectors ***r*** and ***c***, respectively. Singular value decomposition (SVD) [43] of the weighted matrix produces:

[4]
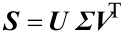


From the above SVD, the following coordinate matrices can be obtained:

[5]
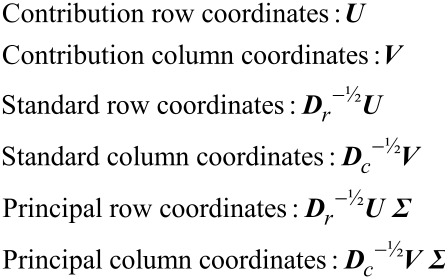


Based on the coordinates provided by LRA, various biplots can be constructed to represent treatments (samples) and bacterial taxa (variables) together. For example, principal row and standard column coordinates can be displayed (using the first two columns of the coordinate matrices) jointly as a row-principal biplot, while the combination of standard row and principal column coordinates yields a column-principal biplot. When there are many components (e.g., bacterial taxa) a convenient alternative is to derive a contribution biplot by combining standard row and contribution column coordinates or contribution row and standard column coordinates [36]. It is noted that LRA requires the compositional data matrix to be strictly positive. However, a few zeros could remain in the compositional data matrix even after the removal of the bacterial taxa that are identified as treatment insusceptible. In the present work, for a given taxonomic level, the remaining vanishing relative abundances of bacterial taxa was substituted by half of the smallest non-zero value in the complete data (before the removal of treatment insusceptible bacterial taxa) [36], followed by a rescaling step to close the data again (i.e., the relative abundance sums to unity for each treatment).

### Multidimensional scaling analysis

Multidimensional scaling (MDS) analysis [32,40] was also conducted for the soil bacterial community dataset with the objective of representing the treatments in a two-dimensional (2D) map while maintaining (as closely as possible) the inter-treatment distance. Unlike LRA, MDS is not subcompositionally coherent [32,36–37] and thus was conducted with the complete dataset (i.e., no bacterial taxa removed) of each taxonomic level (from OTU, genus, …, to phylum). For a given taxonomic level, in order to conduct MDS, distances between treatments need to be calculated first based on their relative abundances. In the present work, Bray-Curtis dissimilarity (BCD), as the most widely used dissimilarity metric in ecological data analyses [32,44], was calculated to quantify the difference between the 15 treatments (in quadruplicate). For raw OTU counts, BCD between two treatments [32] was calculated by:

[6]
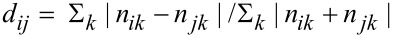


in which *n_ik_* and *n_jk_* represent the *k*-th OTU count for treatment *i* and *j*, respectively. As the OTU counts were converted into relative abundances (*r_ik_* = *n_ik_*/Σ*_k_n_ik_*), the BCD reduces to the regular L_1_ distance [32]:

[7]
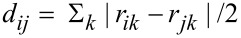


The above L_1_ distance calculation resulted in a 60 × 60 matrix for each taxonomic level since quadruplicates were used for each of the 15 treatments.

Coordinates for plotting the treatments in 2D maps were derived from the L_1_ distance matrices via MDS [32,40] (using the isoMDS function of R package MASS [45]). Since the L_1_ distance is a non-Euclidean distance, the above MDS is referred to as nonmetric MDS (NMDS) [32,40]. The quality of the NMDSs was then quantified by the normalized sum of squared approximation errors known as *stress* [32,40]. In the NMDS established for each taxonomic level there were 60 points, corresponding to the 15 treatments (in quadruplicate). In order to avoid obscureness induced by treatment replicates, reduced NMDSs were developed by using the average-link as the metric to measure the distance between different treatments. The average-link between treatment *S_i_* and *S_j_* was calculated as:

[8]
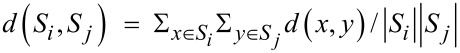


The developed NMDs were converted into biplots by adding vectors to represent bacterial taxa [32]. For a bacterial taxon, the relevant vector was obtained via linear regression of the relative abundance (quadruplicates averaged for the bacterial taxon) on the NMDS coordinates. The vector was formed by the regression coefficients of the NMDS coordinates which then served to indicate the direction the greatest ascent in the regression plane (i.e., gradient vector) [32].

In addition, hierarchical clustering [32,38–39] was carried out based on the L_1_ distance matrices to identify treatments that induced similar impacts on the soil bacterial communities (i.e., the main underlying structure of the MNP soil bacterial community data). Hierarchical clustering successively merges together similar treatments or treatment groups until a single cluster is attained [38–39], providing a dendrogram of hierarchical similarity among the treatments. In the hierarchical clustering, average-link (defined as 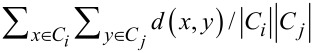 for two clusters *C_i_* and *C_j_*) was used as inter-cluster distance measure since it is robust to outliers [38–39]. An advantage of the hierarchical clustering based on the L_1_ distance matrix is that L_1_ < 0.5 represents a meaningful threshold to cut a dendrogram (hierarchical tree) into suitable meta-clusters, whereas a threshold above 0.5 will lead to clustering of treatments that are more dissimilar than similar [32].

### Consistency analysis of MNP impact

A recently developed distance correlation [41] was used to assess the consistency of MNP impacts on soil bacterial communities summarized in different taxonomic levels. It is noted that each taxonomic level contained a range of taxa, representing a set of vectors where the number of components (i.e., dimensionality) could be much larger than the total treatments (e.g., there are 446 bacterial taxa in genus level and 31,624 in OTU levels). Therefore, conventional correlation analyses such as Pearson correlation [46] and canonical correlation [47] are not applicable for analyzing the consistency between different taxonomic levels. For the above problem, distance correlation is particularly suitable, which quantifies the similarity in treatment distance for different taxonomic levels. In distance correlation analysis [41], a new matrix ***A*** is first constructed from the distance matrix ***a*** that was calculated at taxonomic level T_A_ as 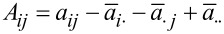, in which 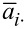, 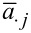 and 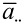 are the means of the *i*-th row, *j*-column, and the entire matrix ***a***, respectively. Similarly, another matrix ***B*** can be derived from the distance matrix ***b*** calculated at taxonomic level T_B_. The distance variances for taxonomic level T_A_ and T_B_ along with their distance covariance can be defined as:

[9]
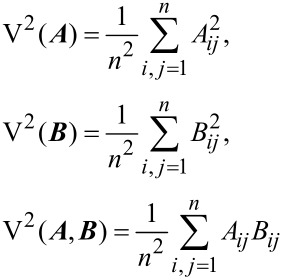


where *n* identifies the dimensionality of matrix ***A*** and ***B***. Accordingly, the distance correlation between taxonomic level T_A_ and T_B_ is given by:

[10]
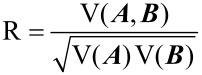


An important property of the above distance correlation is that it becomes zero if and only if the random variables (e.g., different taxonomic levels) are statistically independent [41].

## Results and Discussion

### Bipartite graphs between MNP treatments and bacteria responses

**Figure 2:**
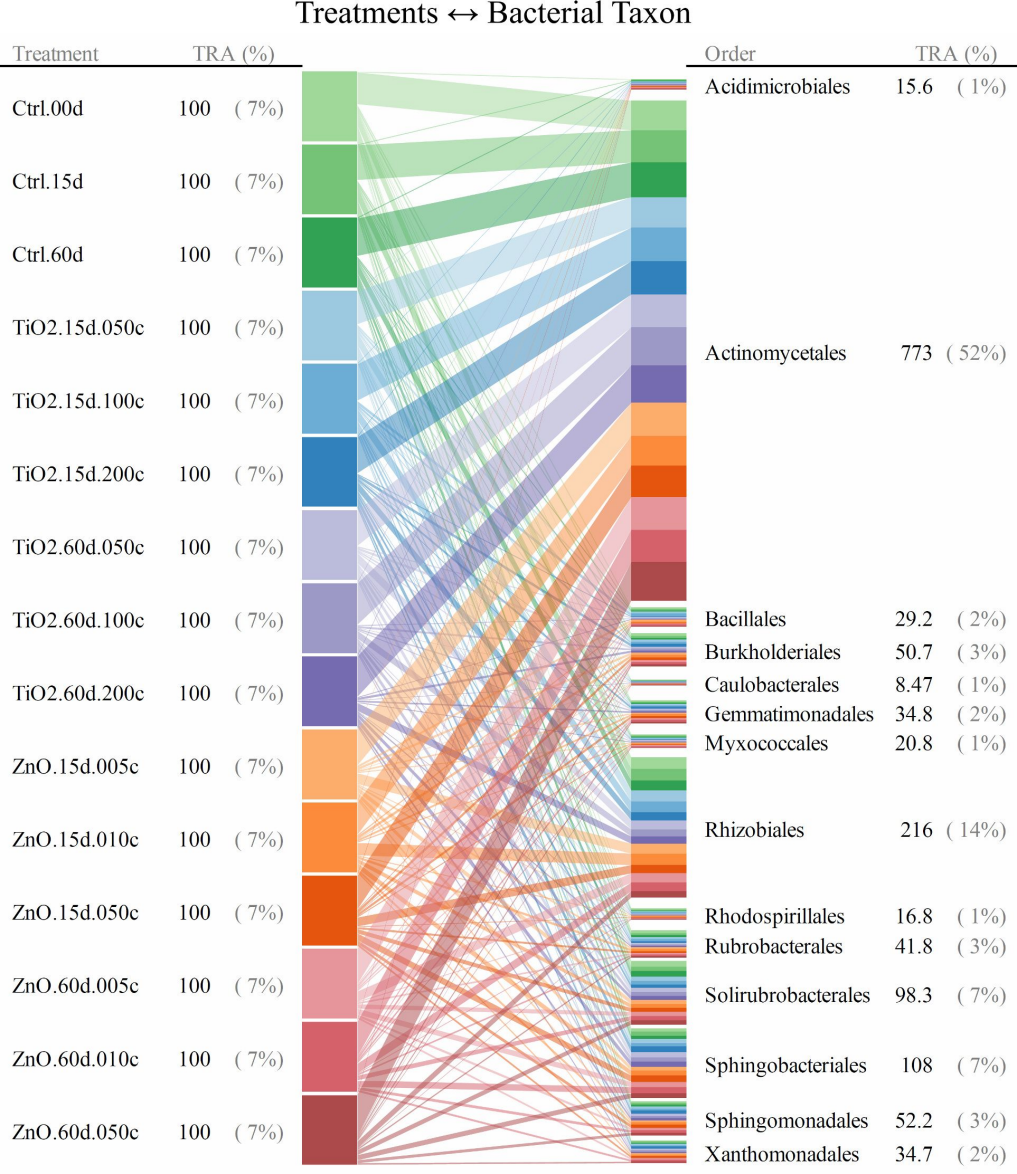
Bipartite graph for MNP-bacteria interrelationships at order level. Soil bacteria taxa identified for the above graph are for the 95th–5th percentile range ≥ 10/*n* (*n* denotes the total number of bacterial taxa at a given taxonomic level) in relative abundance. TRA denotes total relative abundance. The treatments are labelled as “--.##d.##c”, where “--” identifies the treatment type (i.e., TiO_2_ MNP, ZnO MNP, or control), “##d” denotes exposure time of ## day, and “##c” represents exposure dose of ##×10^−2^ mg/g (soil).

**Figure 3:**
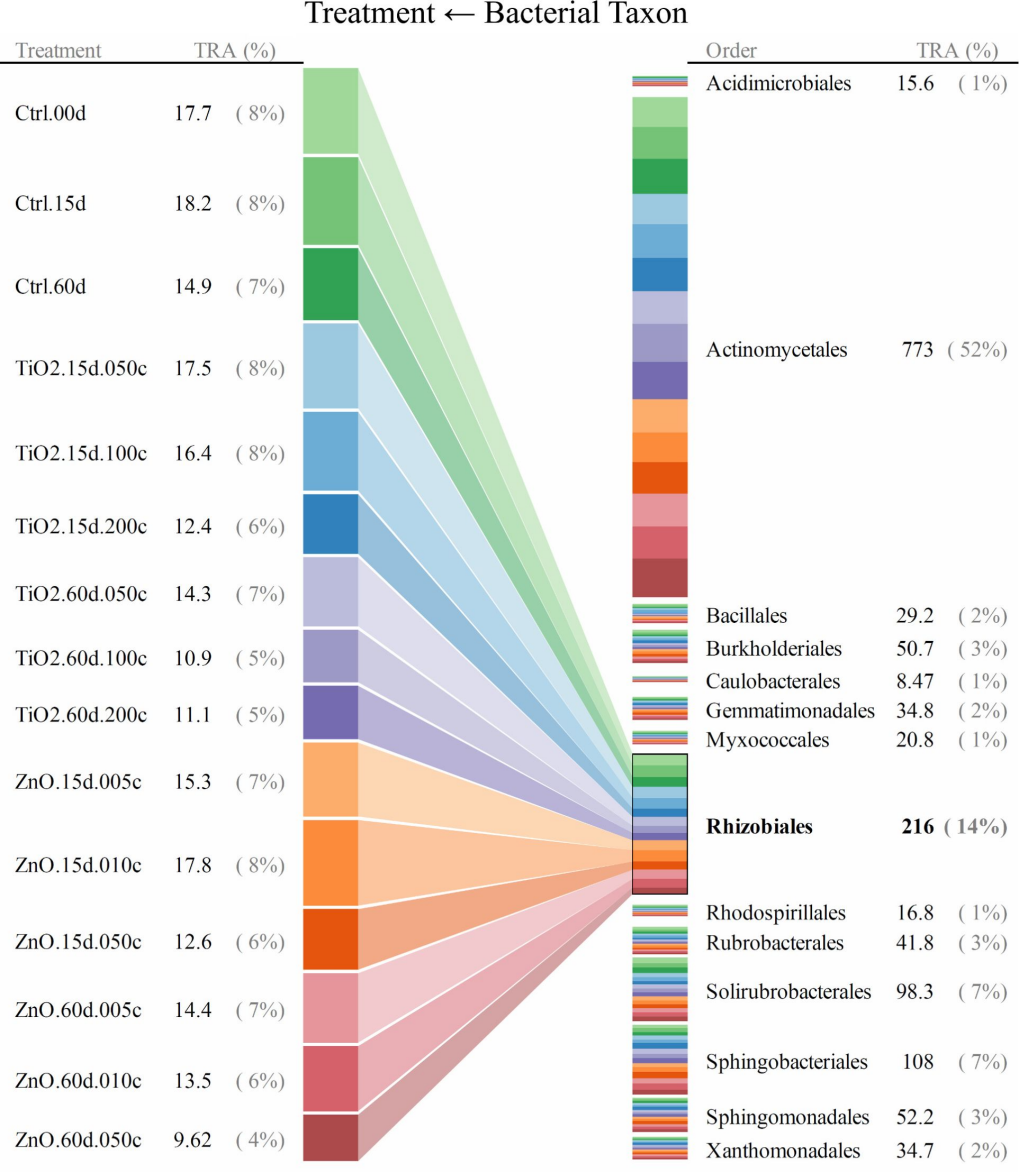
Bipartite graph for bacterial taxon → MNP treatment at order level. Soil bacteria taxa identified for the above graph are for the 95th–5th percentile range ≥ 10/*n* (*n* denotes the total number of bacterial taxa at a given taxonomic level) in relative abundance. TRA denotes total relative abundance. The treatments are labelled as in Figure 2.

**Figure 4:**
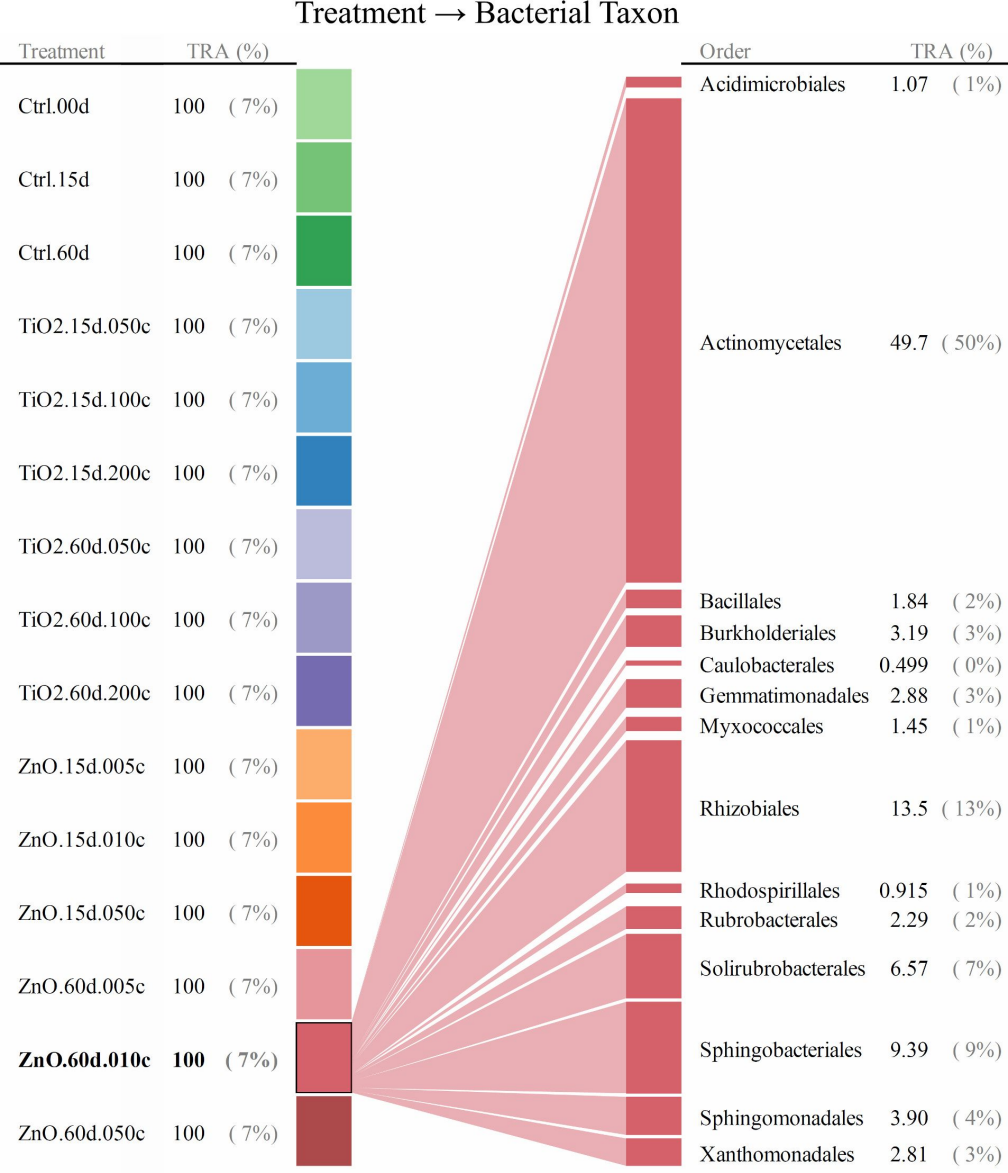
Bipartite graph for MNP treatment → bacterial taxon at order level. Soil bacteria taxa identified for the above graph are for the 95th–5th percentile range ≥ 10/*n* (*n* denotes the total number of bacterial taxa at a given taxonomic level) in relative abundance. TRA denotes total relative abundance. The treatments are labelled as in Figure 2.

**Figure 5:**
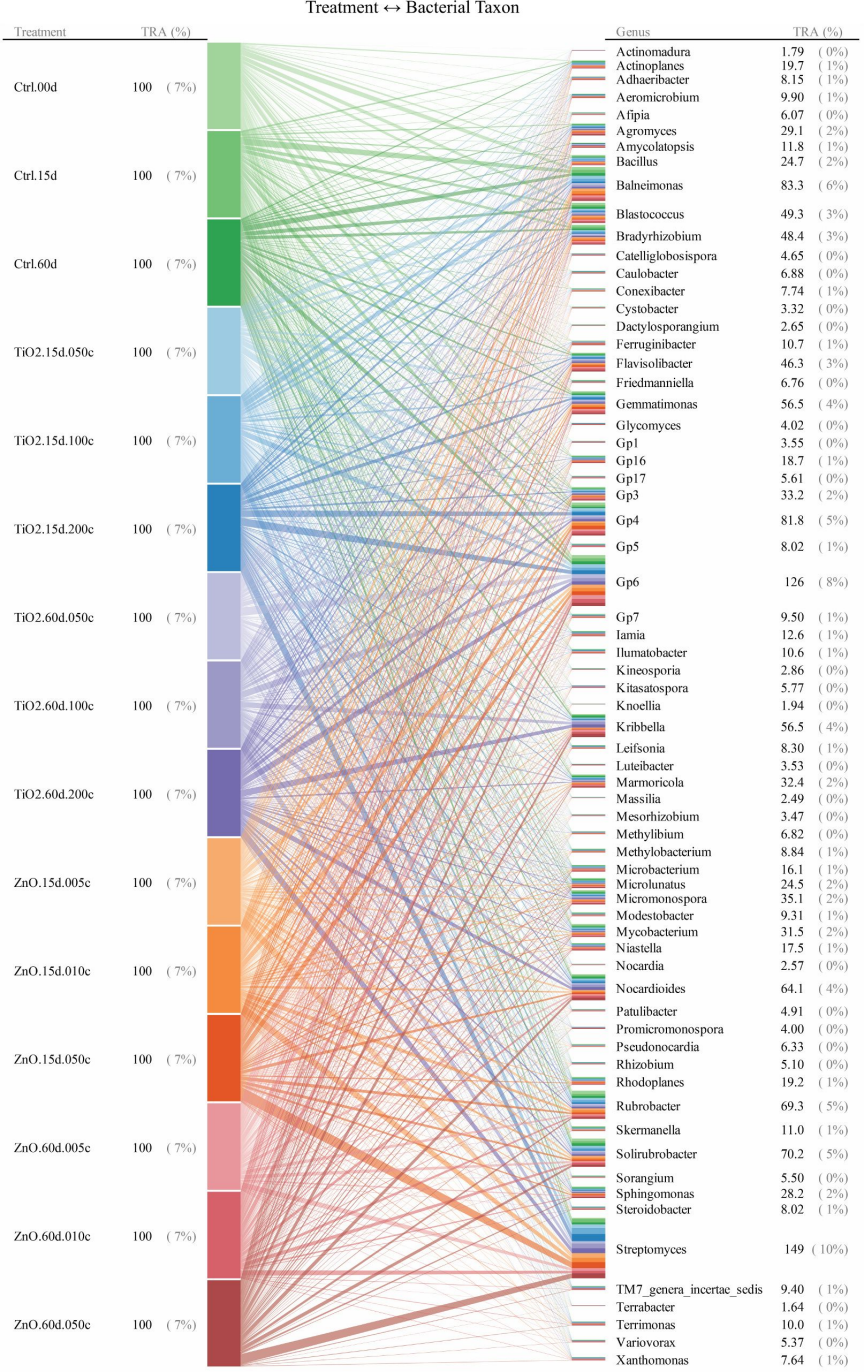
Bipartite graphs for MNP-bacteria interrelationships at genus levels. At genus level, soil bacteria taxa were identified according to an increased threshold of 95th–5th percentile range ≥ 50/*n* (*n* denotes the total number of bacterial taxa at genus level) to avoid cluttering the bipartite graph. TRA denotes total relative abundance. The treatments are labelled as in Figure 2.

**Figure 6:**
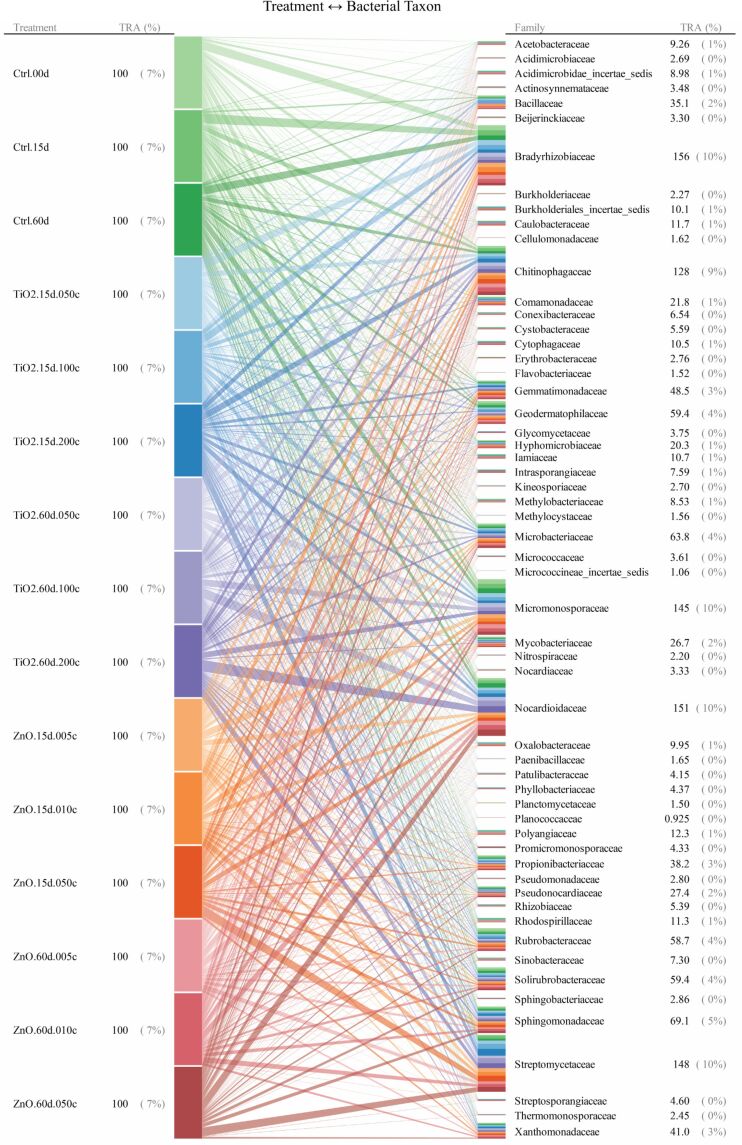
Bipartite graphs for MNP-bacteria interrelationships at family levels. Soil bacteria taxa identified for the above graph are for the 95th–5th percentile range ≥ 10/*n* (*n* denotes the total number of bacterial taxa at a given taxonomic level) in relative abundance. TRA denotes total relative abundance. The treatments are labelled as in Figure 2.

**Figure 7:**
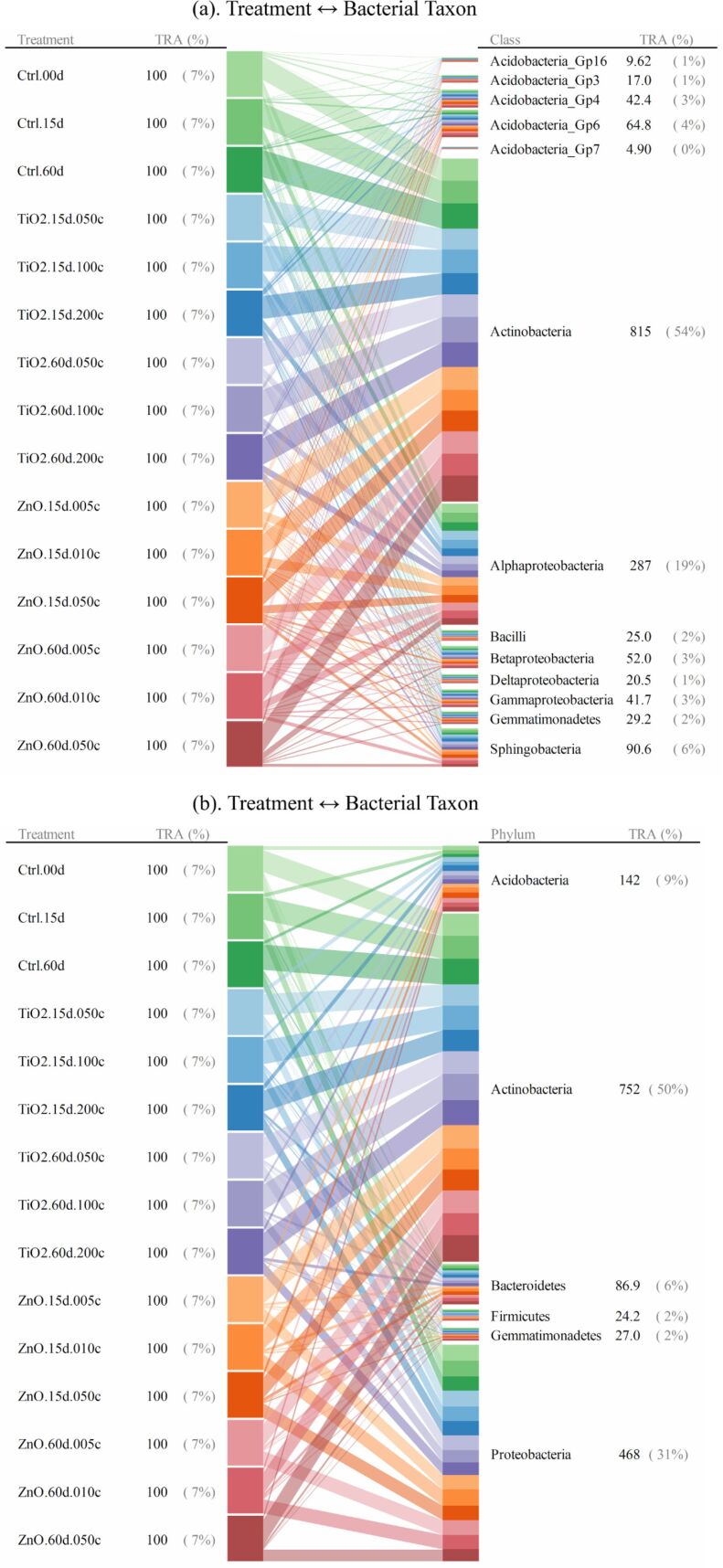
Bipartite graphs for MNP-bacteria interrelationships at (a). class, and (b). phylum levels. Soil bacteria taxa identified for the above graph are for the 95th–5th percentile range ≥ 10/*n* (*n* denotes the total number of bacterial taxa at a given taxonomic level) in relative abundance. TRA denotes total relative abundance. The treatments are labelled as in Figure 2.

For taxonomic levels from genus to phylum, soil bacterial taxa for which the range of 95th–5th percentile with respect to relative abundance (across all the quadruplicated treatments) was no less than 10/*n* (*n* denotes the total number of bacterial taxa at a given taxonomic level) were identified as treatment susceptible. Interrelationships between the 15 treatments and the responses (quantified as relative abundance) of bacterial taxa were illustrated as the bipartite graphs [33–35] established in Figure 2, Figure 3 and Figure 4, as well as Figure 5, Figure 6 and Figure 7. In the bipartite graphs (Figures 2–7), the relative abundances of the soil bacterial taxa identified as treatment susceptible were re-closed (i.e., rescaled such that the relative abundances sums up to unity for each treatment), and then averaged for the quadruplicate of each treatment. It is also noted that, for the genus level, the threshold of 95th–5th percentile range was increased to 50/*n* (where *n* = 446 denotes the total number of bacterial taxa at genus level) in order to avoid cluttering the bipartite graph.

In the bipartite graphs (Figures 2–7), soil bacterial taxa identified as treatment susceptible are denoted by the bars (nodes) on the right side, with the bar height proportional to their total relative abundance over the 15 treatments. For example, *Actinomycetales* is abundant in all the 15 treatments with an average relative abundance of 52% (Figure 2), while, for a specific treatment with ZnO MNPs at the dose of 0.1 mg/g (soil) and exposure time of 60 days, its relative abundance is 50% (Figure 4). Each taxon bar is further split into sub-bars representing its distribution (in terms of relative abundance) across the 15 treatments. The bars on the left side of the bipartite graphs (Figures 2–7) identify the 15 treatments with the bar height indicating the total relative abundance of the taxa identified for the treatments. In the present work, such total relative abundance was 100% for each treatment since the soil bacterial taxa identified as treatment susceptible were re-closed.

The established bipartite graphs can be useful for inspecting soil bacterial taxa that are susceptible to MNPs along with their relative abundance for each treatment. For example, the bipartite graph (Figure 2) for order level shows that only 14 of the 53 bacterial taxa were identified as treatment susceptible, based on the threshold of 95th–5th percentile range ≥ 10/*n* in relative abundance. It is also noted that relative abundances of the above order bacterial taxa vary significantly from 1% to 52%. Moreover, bipartite graphs (Figures 2–7) allow bidirectional exploration of the soil bacterial community data for detailed information about a specific treatment (i.e., bacterial taxon → treatment) or a taxon at different taxonomic levels (i.e., treatment → bacterial taxon). For example, in the direction of bacterial taxon → treatment, focusing the bipartite graph of order level on *Rhizobiales* (Figure 3) revealed that, compared to the controls, the exposure to high TiO_2_ (2.0 mg/g (soil)) or ZnO (0.5 mg/g (soil)) MNP doses for 15 and 60 days reduced the relative abundance of *Rhizobiales* by up to 32% and 35%, respectively. Such relative abundance reductions of *Rhizobiales* indicate that the two MNPs at high dose could stress the *Rhizobiales*. Studies have reported that *Rhizobiales* is an important order taxon containing N_2_-fixing bacteria that are able to symbiotically associate with legume roots to fix atmospheric N_2_ into ammonium for plant growth [48]. One can also explore the effect of treatment on bacterial taxa (treatment → bacterial taxon). For example, the relative abundances of the 14 order taxa displayed in Figure 4 illustrates treatment with ZnO MNPs at the dose of 0.1 mg/g (soil) and exposure time of 60 days, showing that *Actinomycetales* and *Caulobacterales* are the bacterial taxa of the highest (49.7%) and lowest (0.5%) relative abundance, respectively. The above bidirectional exploration using bipartite graphs can be conducted along the taxonomic hierarchy (Figures 5–7) to identify informative MNP-bacteria interrelationships at different levels (e.g., drill down to genus level or roll up to phylum level).

### Contribution biplots generated by log-ratio analyses

**Figure 8:**
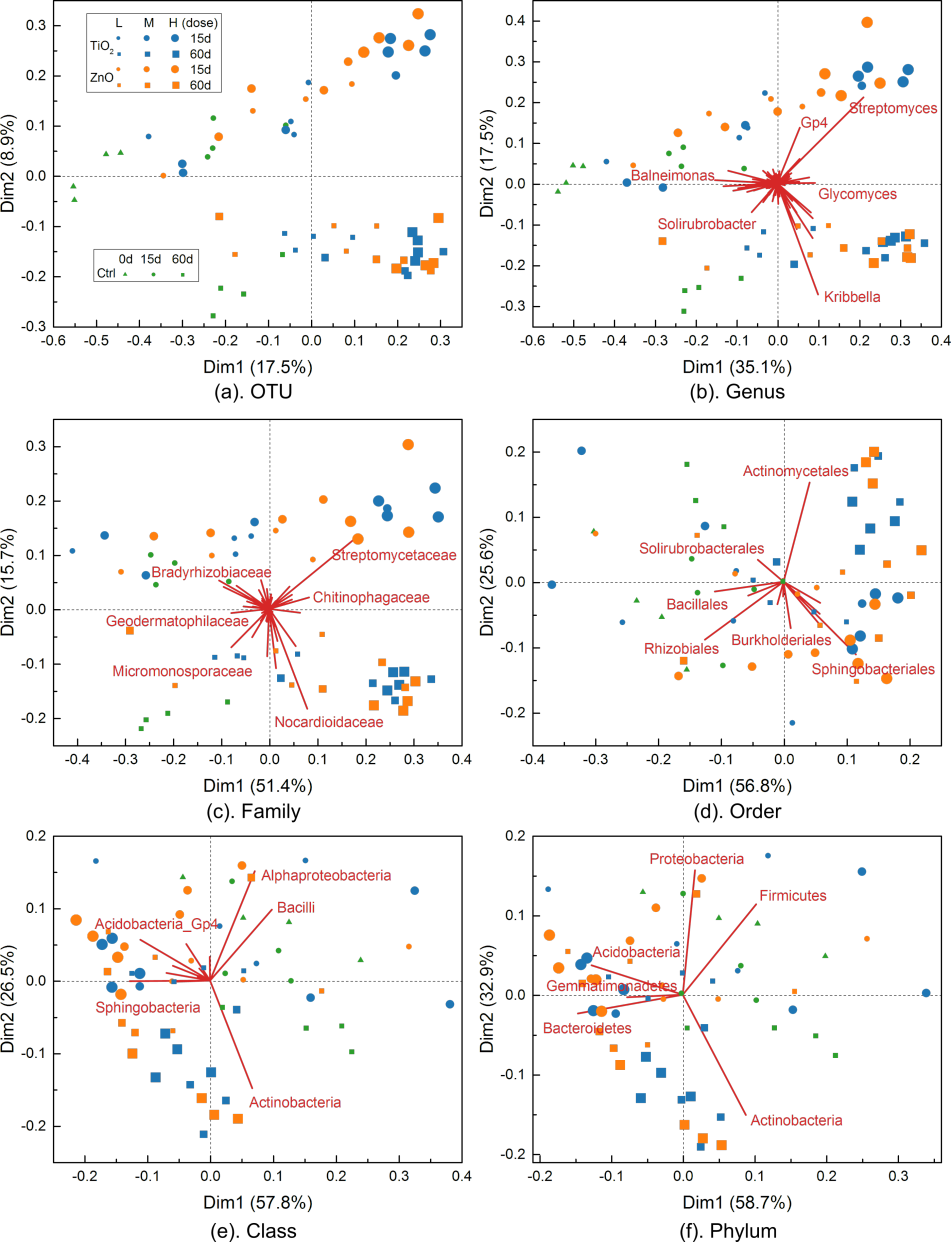
Contribution biplots generated by log-ratio analyses for taxonomic levels from OTU to phylum. The total variance in the complete datasets as accounted by the two principal row coordinates (dim1 and dim2) is provided in the appended parentheses. The contribution vectors (bacterial taxa) were scaled to fit into the scatter plots of the treatments. For the treatments (TiO_2_ and ZnO MNPs and controls (Ctrl)), the exposure time is denoted by “##d” with “L”, “M”, “H” corresponding to doses of 0.5, 1.0, 2.0 mg/g (soil) and 0.05, 0.1, and 0.5 mg/g (soil) for TiO_2_ and ZnO MNPs, respectively. The contribution vectors are omitted for the plot of OTU level to avoid cluttering the plot.

Results of the log-ratio analysis (LRA) [32,36–37] for the soil bacterial community dataset are illustrated in the contribution biplots [32,36] given in Figure 8, which display treatments and bacterial taxa jointly in the same maps. In a contribution biplot (Figure 8), the treatments (samples) are displayed as scatter points using the first two principal row coordinates (i.e., dim1 and dim2) provided by LRA, while the bacterial taxa contributions (variables) were added as vectors (from the origin) scaled to fit into the same range of the principal row coordinates. The scatter plots maintain the distance between different treatments in the complete datasets to a reasonable approximation. The vectors, on the other hand, are indicative of both the contribution (variance across all the treatments) of the bacterial taxa (via vector length) and the correlations between them (via angles between the vectors).

**Figure 9:**
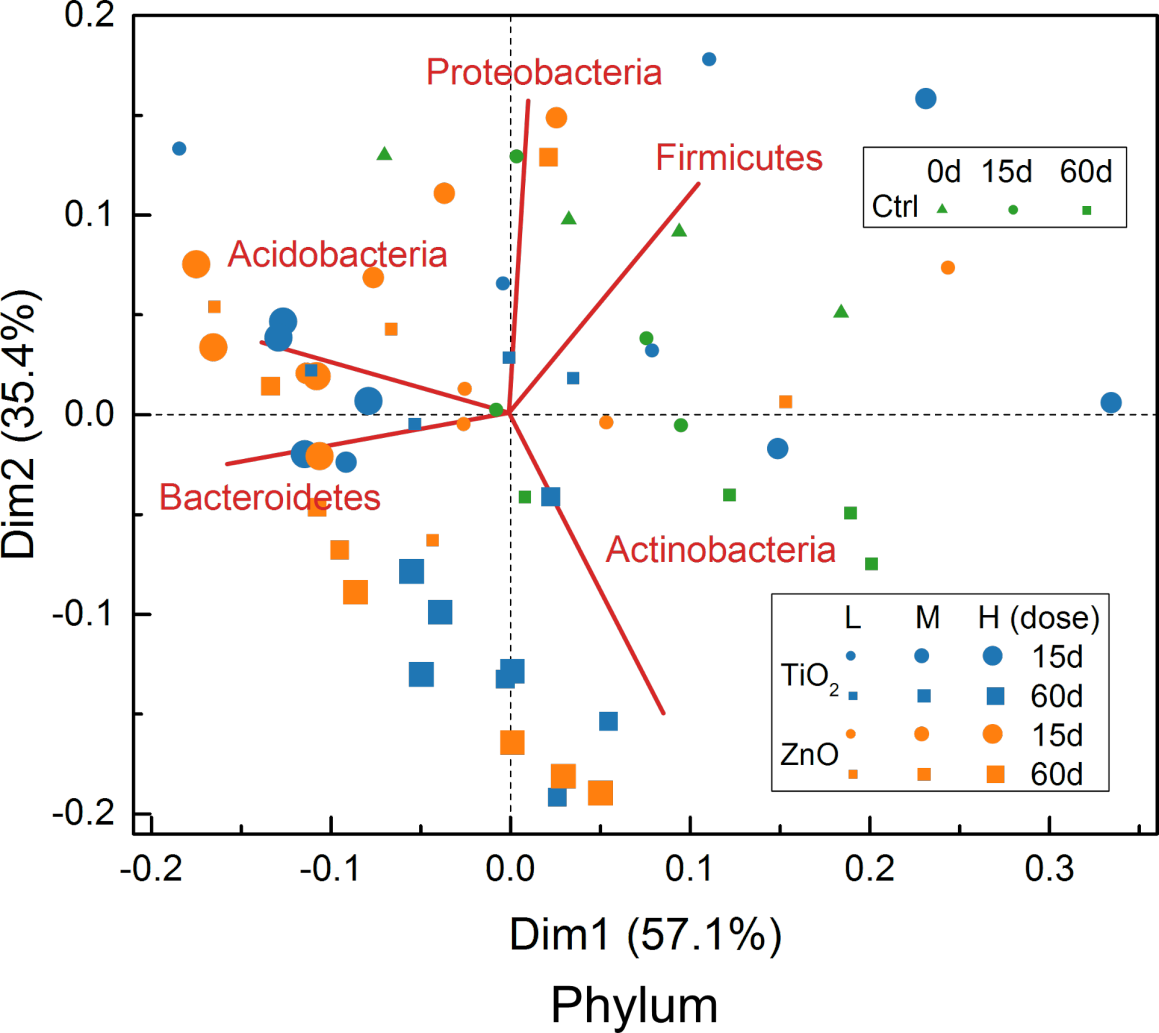
Contribution biplot for phylum level with *Gemmatimonadetes* removed. The treatments are labelled as in Figure 8.

The above configuration of biplots (Figure 8) that display bacterial taxa according to their contributions (variances) to the principal row coordinates allows a visual separation of determinant ones from the large number of bacterial taxa. The correlations between bacterial taxa can be readily inferred from the biplots (Figure 8) along with their contribution to treatment separation. For example, a number of bacterial taxa of significant contribution (vectors of large length) to treatment separation are outlined in each biplot (Figure 8). It is noted that, for order level, *Rhizobiales* is a primary bacterial taxon that separates TiO_2_ and ZnO MNPs from the controls at the high dose. The biplot for order level (Figure 8d) demonstrate that the MNP treatments at high dose had lower relative abundances of *Rhizobiales* compared to controls. The above observation (Figure 8d) is consistent with the bipartite exploration result of order level (Figure 3). Moreover, due to the subcompositional coherence property of LRA [32,36–37], the removal of some bacterial taxa will not change the correlations between the remaining bacterial taxa. For example, the biplot for phylum level remains essentially the same with (Figure 8f) or without (Figure 9) the *Gemmatimonadetes*.

**Figure 10:**
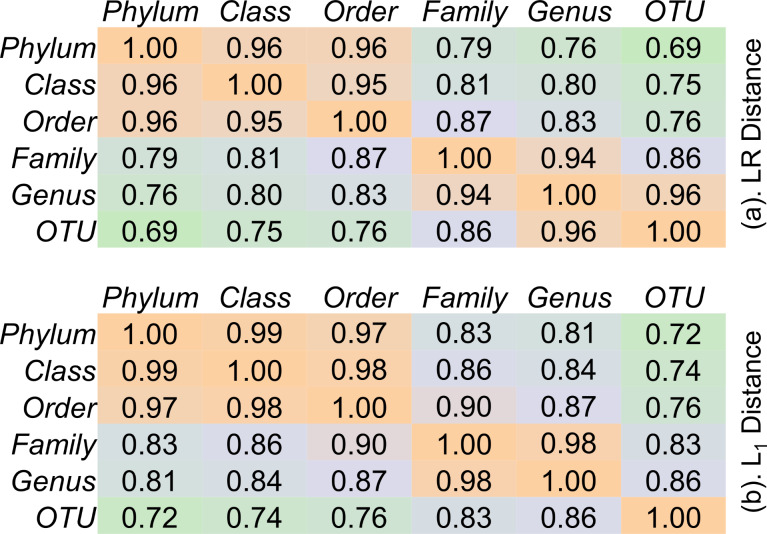
Distance correlation between taxonomic levels from OTU to phylum using (a) log-ratio (LR) distance and (b) L_1_ distance.

The biplots given in Figure 8 also provide useful information regarding the main underlying structures in the soil bacterial community dataset. For example, the biplots for OTU, genus, and family levels (Figure 8a–c) demonstrate that there are two groups of MNP treatments (corresponding primarily to 15 days and 60 days exposure, respectively) separated from the controls. However, as the taxonomic hierarchy increases to order, class, and phylum levels (Figure 8d–f), the treatments are more dispersed (less separable). This indicates that the above taxonomic levels are too high to differentiate the impact of MNPs on soil bacterial communities. In other words, family, as the highest taxonomic level that maintains the main underlying structure of the soil bacterial community data, could be a suitable taxonomic level for MNP impact assessment. Indeed, the distance correlation (Figure 10a) calculated between log-transformed relative abundance of bacterial taxa at different taxonomic levels revealed that the six bacterial taxonomic levels can be divided into two groups of high consistency. The first group contains phylum, class, and order levels with average distance correlation of 0.96, while family, genus, and OTU formed a second group of average distance correlation of 0.92. Compared to the high intra-group consistencies, the average distance correlation between the two groups dropped to 0.78. The above distance correlation analysis again suggests that family could be a suitable taxonomic level for MNP impact assessment as it is the highest taxonomic level of good consistency to the OTU level. The distance correlation analysis (Figure 10a) also indicates that, in general, levels closer in the taxonomic hierarchy are more consistent with each other. Finally, it is also noted that, the two principal row coordinates (i.e., dim1 and dim2) of the biplots for OTU, genus, and family levels (Figure 8a–c) account for <80% of the total variance in the complete datasets (which can be considered as the information preserved by the biplots). The above explained variance increased to >80% in the biplots for order, class, and phylum levels, indicating that the inter-treatment distances were closely maintained in these biplots [32].

### Multidimensional scaling maps

**Figure 11:**
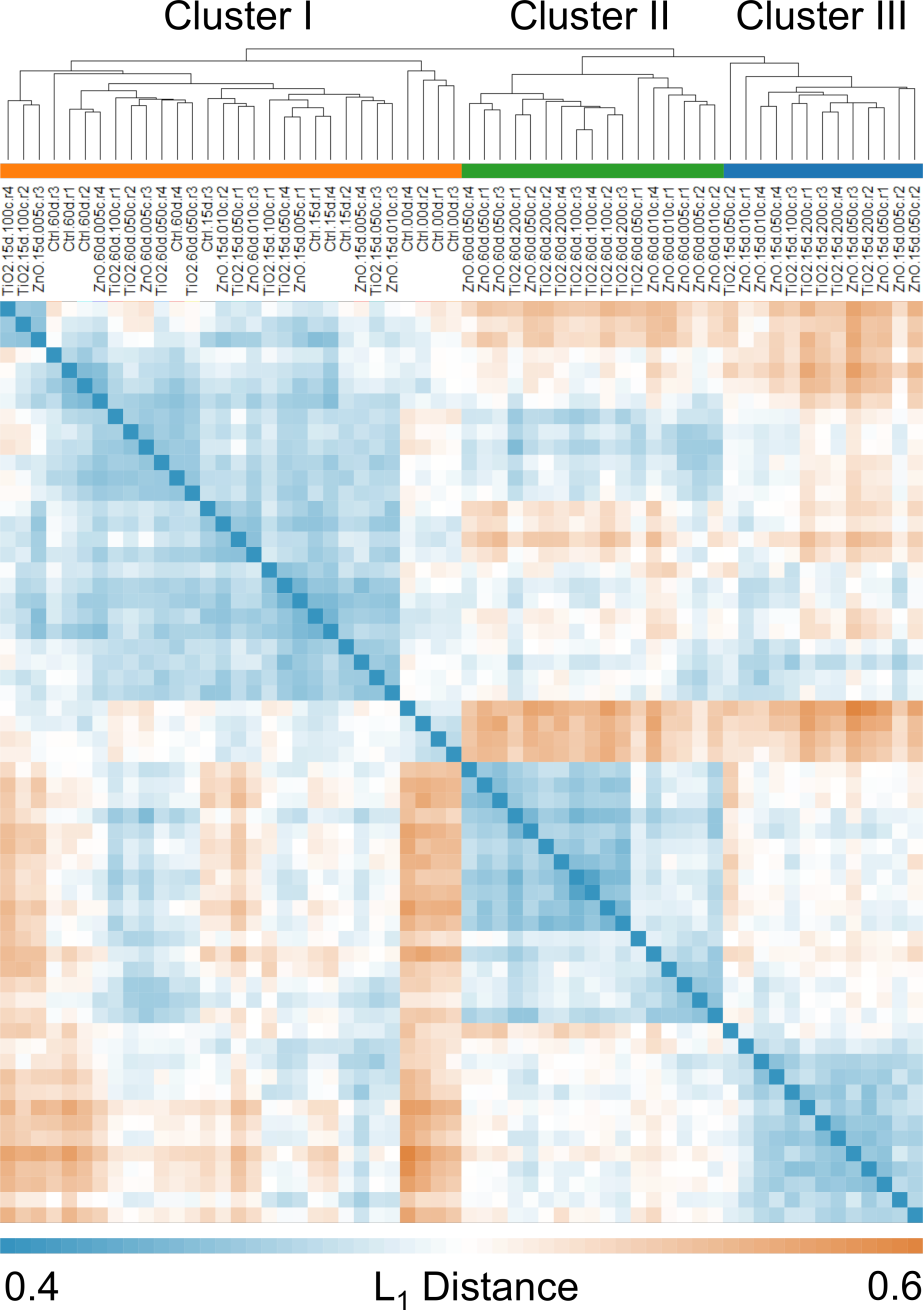
Clusters of treatments obtained via hierarchical clustering based on their L_1_ distances calculated at OTU level. Three meta-clusters were identified according to the recommended threshold of L_1_ < 0.5 [34]. The treatments are labelled as in Figure 2 with an additional “.r#” identifying different replicates.

The L_1_ distance matrix calculated for the 15 treatments (in quadruplicate) at the OTU level is illustrated in Figure 11 as a hierarchically clustered heatmap [32,38–39] established using average-link [32,38–39]. According to the recommended threshold of L_1_ < 0.5 [32], three meta-clusters were identified from the heatmap with Cluster II and III mainly comprised of MNPs exposed for 15 and 60 days and Cluster I formed by the remainder (Figure 11). Characterization of Cluster II and III by exposure time is consistent with the contribution biplot for OTU level (Figure 8a) and previous studies [18–19] that also demonstrated significant impact of exposure period on soil bacterial communities. In addition, all high doses of TiO_2_ (2.0 mg/g (soil)) and ZnO (0.5 mg/g (soil)) MNPs are found in Cluster II and III, while all controls are found in Cluster I (Figure 8), indicating that both MNPs altered soil bacterial communities at relatively high dose.

**Figure 12:**
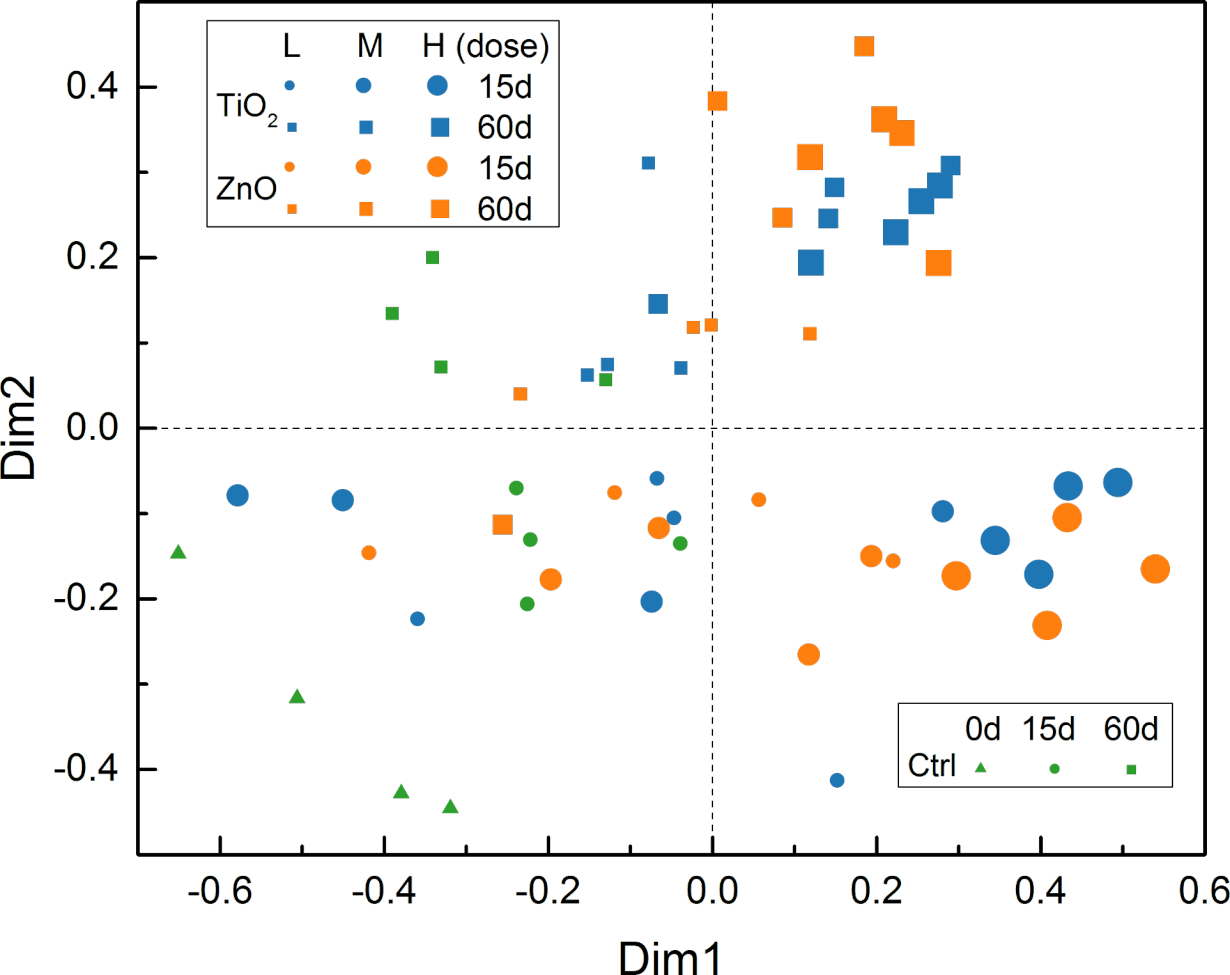
Nonmetric multidimensional scaling (NMDS) for OTU level (*stress* = 14.85%). The treatments are labelled as in Figure 8.

Based on the distance matrix calculated for the OTU level, a 2D map (Figure 12) was established using nonmetric multidimensional scaling (NMDS) for direct presentation of inter-treatment (in quadruplicate) distances. The NMDS established for the OTU level (Figure 12) agrees well with the hierarchical clustering result (Figure 11) with the treatments in Cluster II and III located mainly in the first and fourth quadrants, while the treatments contained in Cluster I are scattered in the second and third quadrants. In addition, the NMDS (Figure 12) further demonstrates that there is large variance within the replicates of each treatment, which obscures the inter-treatment distance relationships. The NMDS for OTU level (Figure 12) is also similar to the contribution biplot (Figure 8a) generated for the same level. Although the above NMDS (Figure 12) had a good *stress* of 14.85% [49]; however, *stress* is usually an over-optimistic measure of preserved/lost information [32] compared to the percent of explained variance (which is not defined for NMDS).

**Figure 13:**
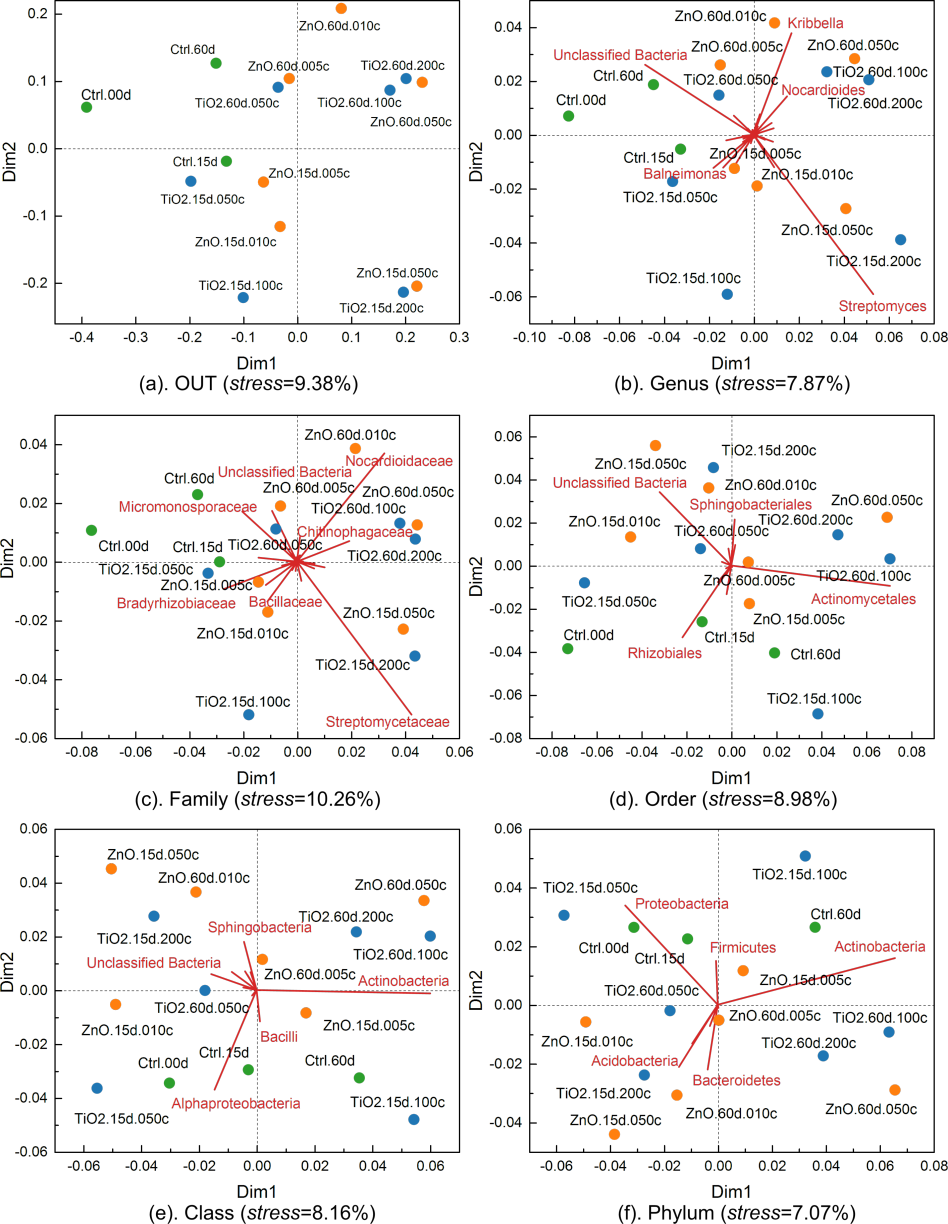
Simplified nonmetric multidimensional scaling (NMDS) for taxonomic levels from OTU to phylum. The gradient vectors of bacterial taxa were scaled to fit into the scatter plots of the treatments. The gradient vectors are omitted for the plot of OTU level to avoid cluttering the plot.

The obscureness caused by the quadruplicate of each treatment is avoided in the NMDS using the average-link of L_1_ distance between different treatments (Figure 13). Without the interference of replicates, the simplified NMDS clearly shows that the high dose of ZnO (0.5 mg/g (soil)) and TiO_2_ MNPs (2.0 mg/g (soil)) have significant impacts on soil bacterial communities at the OTU level (Figure 13a) as they are distant from the controls. Similar behavior of the ZnO and TiO_2_ MNPs is also observed in the simplified NMDSs (Figure 13b,c) established for the genus and family levels. However, as the taxonomic hierarchy increased to order, class, and phylum levels, the treatments (controls and MNPs) disperse and mix with each other on the NMDSs (Figure 13d–f), signifying that the taxonomic levels are too high to differentiate the impact of MNPs on soil bacterial communities. The above observations with the NMDSs are consistent with those from the contribution biplots (Figure 8) generated by LRA. In addition, the distance correlations calculated between the six different taxonomic levels based on L_1_ distance (Figure 10b) are also similar to those obtained based on the log-transformed relative abundance of bacterial taxa. In the NMDSs, a number of bacterial taxa of significant gradients (vectors of large length) are outlined (Figure 13), indicating that their relative abundance varies significantly across the treatments [32]. However, these gradient vectors are not directly related to the contributions of the corresponding bacterial taxa to treatment separation and the NMDSs are not subcompositionally coherent [32,36–37].

## Conclusion

The impact of manufactured nanoparticles (MNPs) on soil bacterial communities was analyzed using a series of visual exploration approaches. The analyzed soil bacterial community dataset contained the counts/relative abundance of a set of hierarchical taxa (at operational taxonomic unit (OTU), genus, family, order, class, and phylum levels) measured for 15 soil treatments with exposure to TiO_2_ (at dose of 0.5, 1.0, and 2.0 mg/g (soil)) and ZnO (at dose of 0.05, 0.1, and 0.5 mg/g (soil)) MNPs for periods of 15 and 60 days or 0, 15, and 60 days without exposure to MNPs (i.e., controls). Bipartite graphs were established to illustrate the inter-relationships between MNPs and responses of bacterial taxa. The bipartite graphs were shown to be useful for identifying, from numerous MNP-bacteria interrelationships, those that reflect significant change in relative abundance of bacterial taxa. Contribution biplots of subcompositional coherence property were generated by log-ratio analysis (LRA) [32,36–37], providing joint displays for the separation (distribution) of treatments and the contribution (variance) of bacterial taxa. The LRA contribution biplots and two-dimensional maps, constructed from the dataset using hierarchical clustering and nonmetric multi-dimensional scaling (NMDS), also demonstrated that high doses of ZnO and TiO_2_ MNPs caused significant compositional changes in soil bacterial communities. The LRA contribution biplots and the simplified NMDSs, together with the distance correlation analysis for the consistency between MNP impacts summarized at taxonomic levels, suggest that family could be a suitable taxonomic level for MNP impact assessment. Utilization of the above visual data exploration approaches can be particularly useful if deployed as a web-based platform for rapid assessment of the impact of MNPs on bacterial soil communities, as well as other ecological systems to guide the development of safe-by-design nanomaterials.
